# Development of a new threshold for pericoronary fat attenuation based on 40 keV virtual monoenergetic images from dual-energy spectral CT

**DOI:** 10.3389/fcvm.2025.1679078

**Published:** 2025-11-19

**Authors:** Jing Chen, Shaoquan Zhou, Min Gu, Kang Li

**Affiliations:** 1 Department of Radiology, Affiliated Hospital of North Sichuan Medical College, Nanchong, China; 2 Department of Radiology, Chongqing General Hospital, Chongqing University, Chongqing, China

**Keywords:** spectral detector computed tomography (SDCT), conventional mixed-energy images (MEI), virtual monoenergetic images (VMI), pericoronary adipose tissue (PCAT), fatattenuation index (FAI)

## Abstract

**Objective:**

To investigate the distinct properties of pericoronary adipose tissue (PCAT) on 40 keV virtual monoenergetic images (VMI) from spectral detector CT (SDCT) and compare them with conventional mixed-energy images (MEI), aiming to establish a novel threshold for the fat attenuation index (FAI) at this energy level.

**Methods:**

We retrospectively analyzed 60 patients who underwent coronary angiography with SDCT. The PCAT around the right coronary artery was selected for FAI assessment. CT attenuation values (minimum, mean, median, maximum), value distribution, background noise, and signal-to-noise ratio (SNR) were compared between 40 keV VMI and MEI within the same volume of interest.

**Results:**

Compared to MEI, 40 keV VMI demonstrated significantly lower minimum, mean, and median CT attenuation values (all *P* < 0.001), a higher maximum value (−30 vs. 54.00 [28.25-89.75] HU), and a broader distribution of CT values. Background noise was similar between the two modalities, while the SNR was significantly higher on 40 keV VMI (*P* < 0.001). The difference in mean CT attenuation was solely associated with photon energy (*P* < 0.001) and was independent of clinical factors. Based on these findings, we established a novel linear regression equation and proposed a new threshold range for FAI at 40 keV VMI.

**Conclusion:**

The attenuation properties of PCAT are fundamentally different on 40 keV VMI compared to conventional MEI. Our findings underscore the critical need for energy-specific interpretation of FAI in spectral CT and provide a foundational step towards its clinical application by proposing a new threshold.

## Introduction

Ischemic heart disease (IHD) is a coronary artery disease (CAD) caused by various factors that lead to coronary artery stenosis or occlusion, resulting in reduced myocardial blood supply and consequently myocardial ischemia, hypoxia, or even necrosis (1). This imbalance between oxygen demand and supply not only impairs cardiac function but also underlies the development of fatal cardiac conditions, including myocardial infarction and sudden cardiac death. According to the Global Burden of Disease report on cardiovascular diseases and risk factors, IHD remains the leading cause of disability and death worldwide (2). The persistent global burden underscores the urgent need for early detection and risk stratification strategies that go beyond anatomical assessment of coronary stenosis, particularly in asymptomatic or minimally symptomatic individuals. Coronary atherosclerosis (CA) is a pathological process characterized by the formation of atherosclerotic plaques and even vascular remodeling in the coronary artery walls, resulting from multiple sequential events, including endothelial dysfunction, lipid accumulation, and inflammation with monocyte recruitment. It is the predominant cause of IHD (3–5). Importantly, this process is not merely a passive lipid deposition but an active, chronic inflammatory response that extends beyond the vessel wall, involving surrounding tissues such as pericoronary adipose tissue (PCAT), which has emerged as a key player in local and systemic vascular inflammation.

PCAT refers to the fat depot that surrounds the coronary arteries. It plays crucial roles in protecting, supporting, and immune responses for the coronary arteries (6, 7). The paracrine regulation between coronary arteries and PCAT during inflammation is bidirectional. This anatomical proximity allows for direct molecular crosstalk between adipocytes and the vascular wall, enabling PCAT to act not only as a structural buffer but also as an active endocrine and paracrine organ that modulates local vascular homeostasis. The coronary artery under inflammatory conditions inhibits preadipocyte differentiation and downregulate adipogenic gene expression (8). As a result, lipid droplet content decreases, leading to a shift in PCAT composition that is detectable on imaging as increased tissue density, an effect that forms the basis for using CT attenuation as a surrogate marker of perivascular inflammation. The fat attenuation index (FAI), derived from conventional mixed-energy CT images (MEI), has been established as a non-invasive imaging biomarker that quantifies this density change and correlates with the extent of coronary inflammation and plaque vulnerability.

Spectral detector computed tomography (SDCT) is based on the principle of conventional MEI, leveraging the fact that different materials exhibit distinct attenuation coefficients at various photon energies, generating their characteristic attenuation curves (9). This energy-dependent attenuation behavior enables material decomposition, allowing for the differentiation of tissues with similar Hounsfield units (HU) on conventional CT, such as fat, water, and iodine. Thereby providing functional insights beyond anatomical morphology. By image reconstruction algorithms, it enables the generation of multiple spectral datasets, such as virtual monoenergetic images (VMI), iodine maps, fat-water separation images, and effective atomic number (Zeff) maps. These outputs provide more comprehensive and material-specific functional imaging information compared to conventional CT. Studies have demonstrated that adipose tissue exhibits the highest signal-to-noise ratio (SNR) and optimal image quality on 40 keV VMI (10–13). The enhanced SNR at 40 keV is primarily attributed to the proximity of this energy level to the k-edge of iodine, which amplifies contrast between iodine-enhanced structures and surrounding fat, making it particularly suitable for evaluating perivascular tissue interfaces. In contrast-enhanced vascular imaging, the contrast between vessels and PVAT is also enhanced at 40 keV, resulting in superior conspicuity (14–17). This improved vessel-fat delineation facilitates more precise localization of coronary plaques and may enhance the detection of subtle inflammatory changes in adjacent adipose tissue, although this potential has not been fully exploited in the context of PCAT assessment. Despite the advantages of 40 keV VMI in fat visualization, there are few studies on PCAT at this energy level. Our study is aimed to address this knowledge gap by investigating the attenuation characteristics of PCAT on 40 keV VMI and defining a new threshold range for the FAI.

## Materials and methods

### Study population

This retrospective study included stable patients with suspected CAD who underwent coronary artery computed tomography angiography (CCTA) using SDCT at Chongqing General Hospital from March to October 2024. Exclusion criteria were as follows: Incomplete clinical or imaging data, or images with suboptimal image quality; presence of calcified plaques in the proximal segment of the right coronary artery (RCA) (18); previous myocardial infarction or surgery; malignant tumors.

### Clinical data

Clinical information was collected for all patients, including sex, age, family history, medical history, and smoking history. Laboratory results included high-density lipoprotein cholesterol (HDL-C), low-density lipoprotein cholesterol (LDL-C), systolic blood pressure (SBP), and diastolic blood pressure (DBP).

Pulse pressure (PP) was calculated as the difference between systolic and diastolic blood pressure:

PP=SBP−DBP(mmHg)

Body mass index (BMI) was computed using the standard formula:

$$\mathrm{BMI}=\frac{\mathrm{Weight}\;(\mathrm{kg})}{\mathrm{Height}\;(m^{2})}$$

### CT equipment and scan parameters

All scans were performed using a dual-layer detector spectral CT (DLCT) system (Philips IQon Spectral CT, Philips Healthcare, Best, the Netherlands). Contrast Injection Protocol: Iodixanol (350 mg I/mL) was administered via the right antecubital vein using a dual-chamber high-pressure injector. The dose was 0.8–1.0 mL/kg, and the injection rate was 5 mL/s. After contrast administration, 40 mL of saline chaser was injected at the same rate. The coronary CTA scan was acquired using a prospectively electrocardiogram-triggered protocol (“Step & Shoot Cardiac”). A bolus tracking technique was used for scan triggering. The descending aorta at the level of the carina was selected as the site for region of interest (ROI) placement. The monitoring scan was initiated 8 s after contrast injection, and scanning was automatically triggered when the attenuation reached a threshold of 90 HU, followed by a 6-second delay before the start of the diagnostic scan. The coronary CTA scan parameters were as follows: the tube voltage was set to 120 kVp with the tube current of 300 mAs; the detector collimation was 64 × 0.625 mm; the pitch was 0.16; the gantry rotation time was 0.27 s.

### Image post-processing

Volumetric data were reconstructed using a standard filtered back-projection algorithm (FBP) with a slice thickness of 0.9 mm and a slice increment of 0.45 mm. Spectral data were simultaneously reconstructed for subsequent analysis. The images reconstructed at 75% of the R-R interval were selected for analysis. Reconstructed image series included 40 keV VMI and conventional MEI. PCAT was segmented using 3D Slicer (version 5.6.2) on conventional MEI, with a threshold range of −190 to −30 HU applied to identify adipose tissue surrounding the RCA (8). The same segmentation was then mapped onto the corresponding 40 keV VMI from the same patient to ensure spatial consistency. Care was taken to exclude the vessel wall, intraluminal structures, and adjacent myocardium during manual editing.

**Figure 1:**
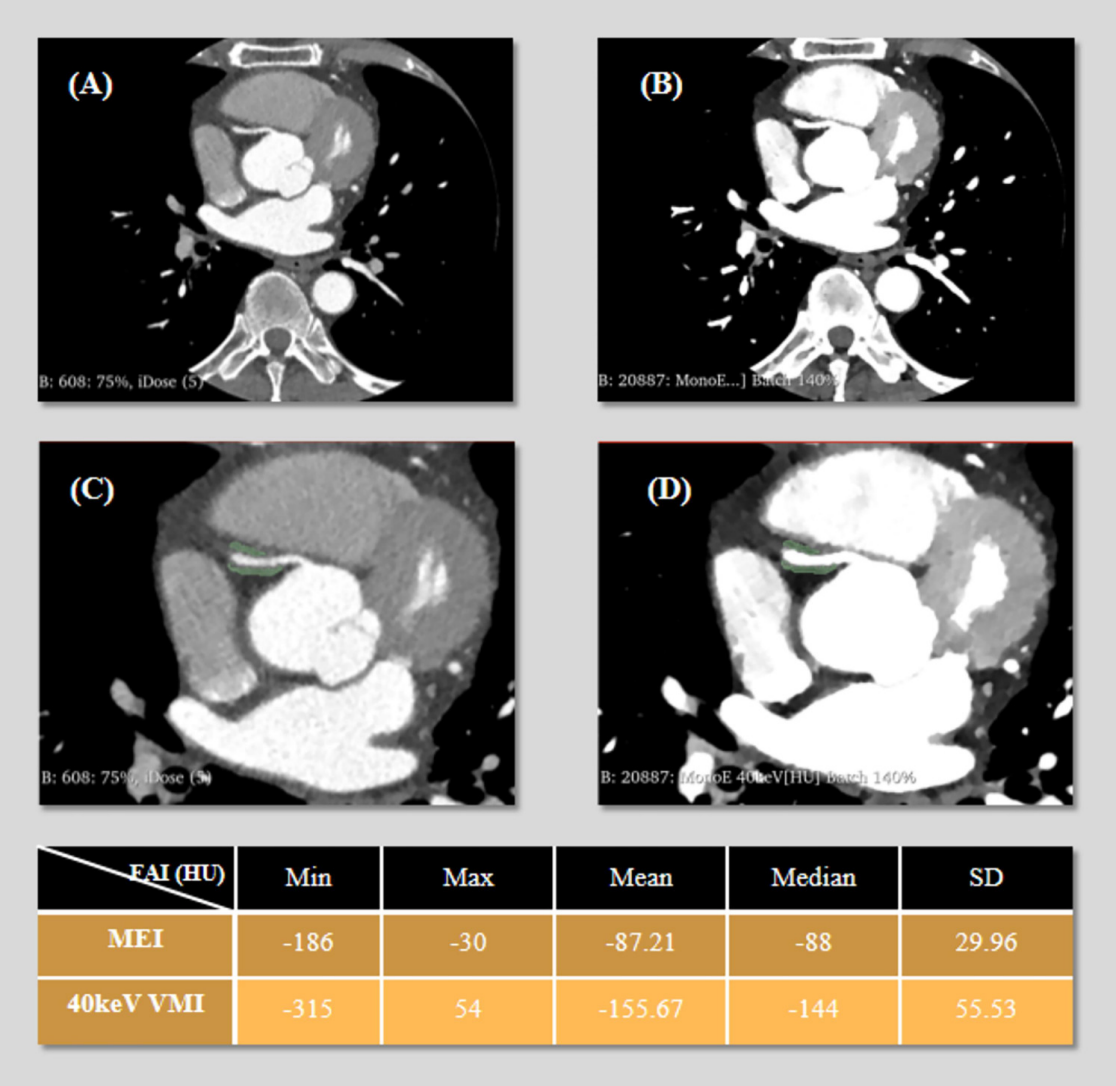
3D Slicer segmentation of pericoronary adipose tissue (PCAT) around the right coronary artery (RCA). The green area represents the segmented volume. **(A)** Axial mixed-energy images (MEI) of the RCA. **(B)** 40 keV virtual monoenergetic images (VMI) at the same level. **(C)** Segment placement on MEI. **(D)** Same region of interest (ROI) on 40 keV VMI. The following table presents the statistical values (minimum, maximum, mean, median, and standard deviation) of fat attenuation index (FAI) measured on both MEI and 40 keV VMI.

**Figure 2:**
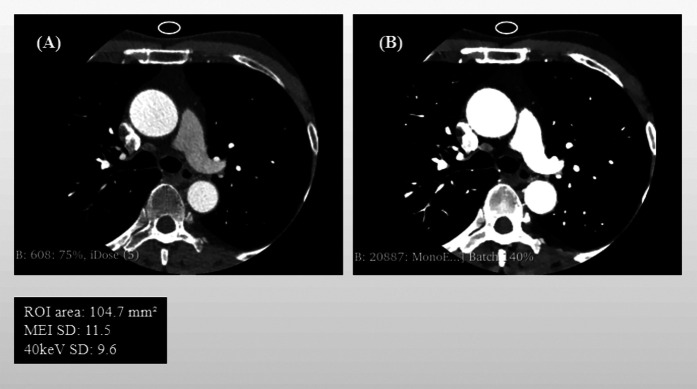
Schematic Illustration of Background Noise standard deviation (SD) and ROI Delineation: mixed-energy images (MEI) vs. 40 keV virtual monoenergetic images (VMI). Panel **(A)** shows the background noise's region of interest (ROI) in MEI, and Panel **(B)** displays the same ROI copied onto the corresponding 40 keV VMI.

Quantitative analysis was performed using the Segment Statistics module to extract CT attenuation metrics, including mean, median, minimum, maximum, and standard deviation (SD), from the pericoronary adipose tissue region on both images types (Figure 1). Background SD: A ROI of approximately 100 mm^2^ was placed in an artifact-free area of air outside the patient's anterior chest wall contour within the scan field of view on the MEI, and the same ROI was copied to the corresponding slice of the 40 keV VMI to measure background noise (Figure 2).

Signal-to-noise ratio (SNR) calculation:

$$\mathrm{SNR}=\frac{\mathrm{Mea}n_{\mathrm{PCAT}}}{SD_{\mathrm{background}}}$$

The post-processing and ROI delineation for both PCAT and background noise measurements were performed by two radiologists, each with over three years of experience in CCTA diagnosis, who received standardized training prior to the study. To evaluate the reproducibility of the measurements, a reliability assessment was conducted. A random sample of 30 patients was selected. One radiologist repeated all ROI delineations (for both PCAT and background noise on both image types) after a one-month interval to assess intra-observer agreement. A second, independent radiologist performed the same delineation procedures on the same set of images to assess inter-observer agreement.

### Statistical analysis

Statistical analyses were performed using IBM SPSS Statistics for Windows, Version 25.0 (IBM Corp., Armonk, NY).Continuous variables that followed a normal distribution are expressed as mean ± standard deviation ($\bar{Mean}\;\pm \;\mathrm{SD}$), while non-normally distributed continuous variables are presented as median and interquartile range [M(Q1,Q3)]. Categorical variables are reported as frequencies and percentages [*n* (%)]. Continuous variables were compared by using the paired t-test, and non-normally distributed variables were compared by using the Wilcoxon signed-rank test; categorical variables were compared by using the independent samples t-test. Intra- and inter-observer agreement for the ROI delineations was assessed using the intraclass correlation coefficient (ICC). An ICC value greater than 0.80 was considered to indicate good agreement.

Correlation analysis was performed using Pearson's correlation for normally distributed variables and Spearman's rank correlation for non-normally distributed variables. Linear regression models were used to evaluate the association between significant predictors and the dependent variables: mean attenuation on 40 keV VMI (40keV_mean) and the difference between mixed-energy and 40 keV mean attenuation (MEI_mean−40keV_mean). Model fit was assessed using the coefficient of determination $\left(R^{2}\right)$. Statistical significance was set at p<0.05.

## Results

A total of 60 patients were included, including 34 men (57%) and 26 women (43%), with a mean age of 63.53±11.26 years. Patient clinical characteristics are summarized in Table 1.

**Table 1:** Patient characteristics.

Characteristics	Value
Age (years ± SD)	63.5 ± 11.3
Male, *n* (%)	34 (57%)
Hypertension, *n* (%)	42 (70%)
PP (mmHg)	52.1 ± 15.12
Diabetes, *n* (%)	18 (30%)
Smoking, *n* (%)	26 (43%)
Alcohol consumption, *n* (%)	24 (40%)
Aspirin use, *n* (%)	11 (18%)
Statin use, *n*(%)	16 (27%)
BMI (kg/m^2^)	25.1 ± 3.03
HDL (mmol/L)	1.23 (1.01–1.50)
LDL (mmol/L)	2.2997 ± 0.79

PP, pulse pressure; BMI, body mass index; HDL, high-density lipoprotein; LDL, low-density lipoprotein; SD, standard deviation.

The reproducibility assessment demonstrated excellent intra- and inter-observer agreement for the measurements of mean PCAT attenuation on both image types and for background noise. Specifically, for the mean PCAT attenuation on conventional MEI, the intra-observer ICC was 0.974 (95% CI: 0.952–0.986) and the inter-observer ICC was 0.962 (95% CI: 0.931–0.979). For the mean PCAT attenuation on 40 keV VMI, the intra-observer ICC was 0.981 (95% CI: 0.965–0.990) and the inter-observer ICC was 0.969 (95% CI: 0.943–0.983). Regarding the standard deviation of background noise, the intra-observer ICC was 0.988 (95% CI: 0.977–0.994) and the inter-observer ICC was 0.978 (95% CI: 0.958–0.989).

The CT attenuation of adipose tissue on 40 keV VMI was significantly lower than that on conventional MEI in terms of minimum, median, and mean values (allP<0.001). The maximum CT value in PCAT higher on 40 keV VMI [median: 54.00 HU (IQR: 28.25–89.75)] compared to conventional MEI. However, it should be noted that the maximum value on MEI was capped at −30 HU due to the segmentation threshold (−190 to −30 HU), which may have artificially limited the observed maximum.

Within the predefined region, the CT value distribution on 40 keV VMI was more dispersed, with a significantly higher SD compared to MEI (P<0.001).There was no statistically significant difference in background noise between the two image types, but SNR was significantly higher on 40 keV images (P<0.001). The measurements are resumed in Table 2.

**Table 2:** Attenuation differences between MEI and 40 keV VMI.

Parameter(HU)	MEI	40 keV VMI	Test statistic	*P*
Min	−173.50 (−186.00, −161.50)	−291.70 ± 45.81	24.97	<0.001
Median	−81.58 ± 13.07	−156.23 ± 31.72	24.16	<0.001
Mean	−81.77 ± 12.03	−153 ± 30.46	23.94	<0.001
Fat SD	27.01 ± 4.37	50.22 ± 10.85	−20.48	<0.001
Max	−30.00	54.00 (28.25–89.75)	–	–
Background Noise SD	13.50 (10.73–17.98)	12.80 (10.33–18.88)	0.73	0.464
SNR	5.74 (4.30–7.64)	11.00 (7.54–15.90)	6.69	<0.001

MEI, mixed-energy images; VMI, virtual monoenergetic images; Min, minimum; Max, maximum; SD, standard deviation; SNR, signal-to-noise ratio. Note: The minimum and maximum values on mixed-energy images were constrained by the segmentation threshold (−190 to −30 HU). Values outside this range were excluded. Therefore, the maximum CT value is not comparable between the two image types and no statistical test was performed.

In correlation analysis, the mean CT attenuation of PCAT on 40 keV VMI was significantly correlated only with the mean attenuation on conventional MEI, showing a positive association (r=0.736,P<0.001). Neither the mean attenuation on 40 keV images nor the difference in mean attenuation between the two image types was significantly associated with patient-specific clinical factors such as age, sex, or comorbidities (Tables 3, 4). Furthermore, background noise (SD) at different energy levels showed no significant correlation with either the mean attenuation on 40 keV VMI or the attenuation difference between the two image types (Table 4).

**Table 3:** Group differences in 40 keV_mean attenuation and the attenuation difference by categorical variables.

Variables	40 keV_mean		MEI_mean-40 keV_mean	
	Mean	*P*	Mean	*P*
Sex				
Male	−147.59	0.108	21.43	0.093
Female	−160.39		24.32	
Hypertension				
Yes	−152.78	0.892	71.13	0.906
No	−153.95		71.90	
Diabetes				
Yes	−153.55	0.945	70.96	0.931
No	−152.95		71.52	
Smoking				
Yes	−144.86	0.066	65.82	0.105
No	−159.45		75.59	
Alcohol consumption				
Yes	−146.67	0.182	66.02	0.145
No	−157.44		74.92	
Aspirin use				
Yes	−147.79	0.524	66.24	0.420
No	−154.33		72.51	
Statin use				
Yes	−154.54	0.831	71.82	0.926
No	−152.62		71.19	

MEI, mixed-energy images.

**Table 4:** Correlation analysis.

Variables	40 keV_mean		MEI_mean-40 keV_mean	
	*r*	*P*	*r*	*P*
Age	−0.101	0.443	0.041	0.758
BMI (kg/m^2^)	0.093	0.481	−0.113	0.390
HDL (mmol/L)	0.015	0.908	−0.005	0.970
LDL (mmol/L)	0.147	0.263	−0.176	0.178
PP	0.121	0.359	−0.135	0.303
40 keV_background noise SD	0.039	0.765	−0.042	0.747
MEI_background noise SD	0.023	0.859	−0.044	0.737
MEI_mean	0.736	<0.001	–	–

BMI, body mass index; HDL, high-density lipoprotein; LDL, low-density lipoprotein; PP, pulse pressure; SD, standard deviation; MEI, mixed-energy images.

A simple linear regression was performed to assess the relationship between the mean CT attenuation of PCAT on MEI and 40 keV VMI. The model was statistically significant [*F*(1, 58) = 68.67, *p* < .001], with an *R*^2^ of 0.542, indicating that 54.2% of the variance in 40 keV mean attenuation was explained by the MEI mean. The unstandardized coefficient was significant (B = 1.864, SE = 0.225, *t* = 8.286, *p* < .001) (Table 5).

**Table 5:** Linear regression model predicting mean attenuation on 40 keV VMI.

Predictor	B	SE	*t*	*P*	*R* ^2^
(intercept)	−0.731	18.588	−0.039	0.969	0.542
40 keV_mean	1.864	0.225	8.286	<0.001	

B, unstandardized coefficient; SE, standard error; *t*, *t*-statistic; *R*², coefficient of determination.

**Figure 3:**
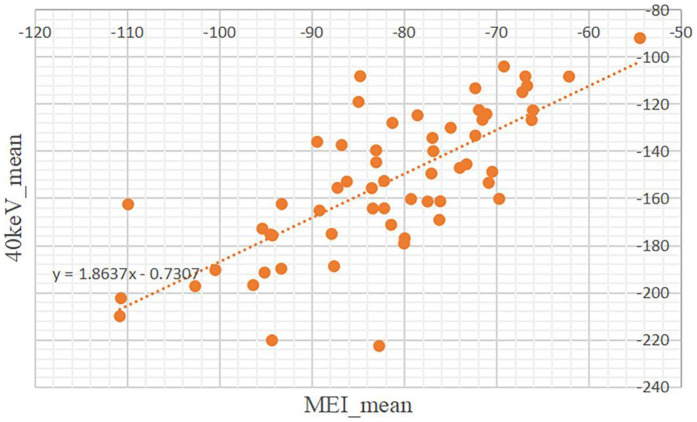
Scatter plot showing the relationship about the mean of CT attenuation values between mixed-energy images (MEI) and 40keV virtual monoenergetic images (VMI). Data points are orange, with a dotted trendline indicating a positive correlation.

The resulting regression equation is: (Figure 3).

$${\hat{y}}_{40\;\mathrm{keV}\_\mathrm{mean}}=1.864x_{\mathrm{MEI}\_\mathrm{mean}}-0.731.$$

Based on the derived regression model, the conventional FAI threshold (−190 to −30 HU) was transformed to estimate the corresponding attenuation range on 40 keV images.

## Discussion

Our study's key findings can be summarized as follows: (1) Within the conventional FAI threshold range of (−190 to −30 HU), the CT attenuation of PCAT on 40 keV VMI was significantly lower, with lower minimum, median, and mean values; (2) The attenuation distribution on 40 keV images was more dispersed, as indicated by a significantly higher SD; (3) Despite no significant difference in background noise, the SNR was significantly higher on 40 keV VMI, suggesting improved image quality for fat visualization; (4) Changes in PCAT attenuation between the two image types were largely independent of patient-specific clinical factors and were primarily associated with photon energy level; (5) Based on the linear relationship between the two image types, we established a regression equation and derived a predicted FAI threshold range of −355 to −57 HU for PCAT segmentation on 40 keV images.

Dual-layer detector spectral CT (DLCT) enables energy-resolved imaging by utilizing a dual-layer detector technology. By exploiting the differential attenuation of x-ray photons in tissues, the system analyzes the signal differences between the top and bottom detector layers to separate low- and high-energy components. Through advanced post-processing, this data is used to generate VMI and other characteristic spectral image series (19). This energy-based decomposition allows for the suppression of beam-hardening artifacts and improved tissue contrast discrimination, particularly at low keV levels where photoelectric effects are amplified. By reconstructing images at optimal energy levels, spectral CT can enhance the visual distinction between materials with similar attenuation properties on conventional imaging, such as soft tissues, fat, and iodine-laden structures.

Iodine exhibits energy-dependent attenuation, leading to pronounced differences in the appearance of materials on spectral CT. *in vivo* measurements from the study by David C. Rotzinger et al. demonstrated that VMI at 40–55 keV enables maintenance or even improvement of the contrast-to-noise ratio (CNR) between vessels and surrounding tissues, including fat, muscle, and bone, despite a 40% reduction in iodine dose and a 50% decrease in injection rate, thereby preserving diagnostic image quality (10). Their corresponding phantom study showed the similar results. Notably, low-keV VMI effectively improved the quantification accuracy of vessel area in a phantom simulating overweight patients (120 kg). In a separate phantom study by Leening P. Liu et al., spectral imaging demonstrated highly consistent quantitative performance for luminal diameters >6 mm, regardless of phantom size or iodine concentration. For smaller vessels (<6 mm), quantitative accuracy improved as phantom size decreased. These studies collectively suggest that spectral CT can achieve superior vessel-to-tissue contrast even under conditions of reduced radiation and contrast agent doses (20). High spatial resolution combined with spectral imaging enables clear visualization of small coronary lesions, such as calcified plaques, even in phantoms simulating overweight patients. The quantitative measurements derived from these images remain consistent across different phantom sizes, suggesting that 40 keV VMI may provide reliable and reproducible characterization of perivascular structures independent of body habitus. Although our study did not investigate the impact of contrast dose or CT acquisition parameters, we acknowledge that these factors may contribute to the observed CT number differences between VMI and MEI. To control for confounding variables, all patients were scanned using the same CT system, identical scanning protocols, and consistent contrast injection regimens, thereby enhancing the comparability of our results.

Although VMI from spectral CT has demonstrated superior contrast resolution and noise properties, particularly at low energy levels, research on PCAT remains predominantly based on conventional MEI. PCAT has been shown to play a crucial role in the development of coronary atherosclerosis. Antonopoulos, A. S. et al. provided a detailed discussion on the pathophysiological mechanisms of PCAT, and their study established the FAI, derived from CTA, as a non-invasive imaging biomarker reflecting inflammatory changes in PCAT (8). Liao Xiyan et al. investigated the predictive value of FAI for acute coronary syndrome (ACS), demonstrating that FAI around the proximal main coronary arteries is an independent predictor of ACS (21). The research by Oikonomou et al. established the prognostic value of FAI, finding that the optimal threshold for perivascular FAI was −70.1 HU, beyond which cardiac mortality increased sharply, and this threshold could identify high-risk individuals with a 5- to 9-fold increased risk of cardiac death (22). Subsequent research further expanded the application dimensions of FAI, the study by Mancio and Oikonomou introduced radiomics technology, revealing that perivascular FAI dynamically changes with acute coronary inflammation, while FRP (radiomics texture features) can capture more permanent structural changes in PVAT, and the combination of both could provide a more comprehensive personalized cardiac risk assessment for each patient (23). These studies collectively demonstrate that from simple density measurements to complex radiomics analysis, PCAT is gradually becoming an important tool for cardiovascular risk assessment. Given that spectral CT enables enhanced fat-vessel contrast and improved tissue characterization at low keV levels, PCAT attenuation could potentially be more accurately captured under energy-resolved imaging conditions.

In a study by Rodriguez-Granillo, G. A. et al., fat depot attenuation was evaluated using spectral CT, demonstrating significant differences in CT values at different energy levels. Specifically, pericoronary adipose tissue surrounding the RCA and the proximal/mid left coronary artery exhibited significantly lower attenuation on 40 keV and 70 keV VMI compared to 120 keV images, in both contrast-enhanced coronary angiographic and delayed-phase scans (11). Notably, no significant association was found between BMI and fat density in that study. Our findings are consistent: the differences in CT attenuation between VMI and MEI were not significantly associated with patient-specific clinical characteristics. This may be, as demonstrated in this study, due to the fact that PCAT changes are primarily driven by paracrine signaling from the vessel wall and strongly influenced by the presence of vascular disease, rather than systemic metabolic or inflammatory states.

Despite growing evidence that pericoronary adipose tissue (PCAT) attenuation differs significantly between VMI and conventional MEI, no study has yet established energy-specific threshold ranges for PCAT segmentation on VMI. Borbála Vattay et al. investigated coronary plaque assessment using SDCT and found significant differences in estimated plaque volume across VMI (12). They emphasized the need for standardized protocols to ensure comparability of results, particularly for low-attenuation non-calcified plaque (LAP) volume, which is currently quantified using fixed HU thresholds. These findings highlight a critical gap in current spectral CT practice: while energy-dependent attenuation effects are well recognized, standardization efforts have focused primarily on plaque characterization, with little attention to the redefinition of fat attenuation metrics. This underscores the necessity of developing VMI-specific reference criteria for PCAT to enable accurate, reproducible, and clinically meaningful quantification in the era of spectral imaging.

To date, only the study by Xujiao Chen et al. (13) has proposed an energy-specific threshold for PCAT segmentation on 40 keV VMI, ranging from −280 HU to −40 HU. This threshold was derived by multiplying the conventional MEI range (−190 to −30 HU) by a conversion factor of 1.44 (24). However, it must be critically noted that this factor of 1.44 was not derived from a principled computational model but was merely calculated as the ratio of the mean PCAT attenuation on 40 keV VMI (181.5 ± 21 HU) to that on non-contrast CT (113.7 ± 9 HU), as reported in the study by Rodriguez-Granillo, G. A. et al. (11). Firstly, the fundamental concept of the FAI was established and validated on contrast-enhanced CTA images, not on non-contrast CT images (8). Secondly, the study did not provide a clear data calculation method nor other methodological justification or validation for this threshold, therefore the proposal of this threshold remains to be further evaluated. Despite the range differs from that derived in our analysis, they similarly reported enhanced detectability of high-attenuation components within plaques at 40 keV.

Notably, their work represents not only a methodological initiative but also an exploratory step toward clinical translation. Based on their redefined threshold, they further demonstrated that PCAT attenuation changes assessed at 40 keV exhibited superior discriminative performance compared to conventional MEI, suggesting that the FAI derived from 40 keV VMI may demonstrates potential as a new non-invasive indicator for assessing plaque vulnerability (13). It must be noted, however, that the threshold derived in our study is based on a cohort of patients with stable coronary artery disease. Therefore, its applicability in patients with acute inflammatory conditions such as acute coronary syndrome remains unknown. In line with these findings, our study will further explore and validate the diagnostic value of PCAT attenuation, using the threshold range derived from our regression model, in the context of cardiovascular disease risk stratification.

Furthermore, in our analysis, we excluded regions adjacent to coronary artery calcified plaques during pericoronary adipose tissue segmentation. This approach was primarily because calcified plaques, due to their high density, are prone to beam-hardening artifacts and partial volume effects, which can significantly interfere with the true CT attenuation values of surrounding adipose tissue. As this measurement bias stems from physical factors rather than biological inflammation of the vessel wall, such regions required exclusion (18). It is noteworthy that coronary artery calcification is a common manifestation of atherosclerosis; in this study, 16 patients were excluded due to significant calcification. Calcification burden, typically quantified by the coronary artery calcium score (CACs), is particularly important for intermediate-risk individuals. It can reclassify cardiovascular disease risk beyond conventional risk factors, proving especially valuable when the estimated risk is near clinical decision thresholds. A higher CACs indicates a greater burden of calcified plaque and is significantly associated with an increased risk of myocardial infarction and death (25). Consequently, we must acknowledge that the 40 keV VMI threshold range (−355 to −57 HU) derived from the current non-calcified patient cohort in this study may not be applicable to vessel segments with calcified lesions. Our subsequent research will be needed to validate this threshold in broader populations, with further stratified analysis based on the degree of calcification.

Beyond these considerations related to calcification, this study has additional limitations. First, it was a single-center, retrospective analysis with a limited sample size (*n* = 60), which may affect the generalizability of the findings. Second, the linear regression model, which forms the mathematical basis for the proposed threshold, explained only 54.2% of the variance in 40 keV mean attenuation. This indicates that factors beyond a simple linear relationship with MEI values influence the PCAT attenuation on VMI. Therefore, while this model provides an essential initial standardization, the derived threshold itself constitutes a proposal that necessitates further validation against clinical outcomes, inflammatory markers, and histopathology to fully establish its biological and clinical significance. Third, due to the fixed segmentation threshold (−190 to −30 HU) applied to conventional mixed-energy images, their maximum CT values were capped at −30 HU. Consequently, the higher maximum values observed on 40 keV images reflect a broader attenuation range rather than true biological differences, preventing direct comparison between the two imaging techniques. These findings still require histopathological validation to determine whether the observed attenuation characteristics represent true adipose tissue or are influenced by adjacent structures.

## Conclusions

In summary, PCAT exhibits lower attenuation levels and improved image quality on 40 keV VMI compared to conventional MEI. The newly defined threshold range established in this study provides the fundamental standardization necessary for quantifying pericoronary inflammation on spectral CT. This advancement holds the potential to improve cardiovascular risk stratification and enhance the prediction of coronary artery disease.

## Data Availability

The original data supporting this study are available from the corresponding author upon reasonable request.
